# The NS segment of H5N1 avian influenza viruses (AIV) enhances the virulence of an H7N1 AIV in chickens

**DOI:** 10.1186/1297-9716-45-7

**Published:** 2014-01-25

**Authors:** Júlia Vergara-Alert, Núria Busquets, Maria Ballester, Aida J Chaves, Raquel Rivas, Roser Dolz, Zhongfang Wang, Stephan Pleschka, Natàlia Majó, Fernando Rodríguez, Ayub Darji

**Affiliations:** 1 Centre de Recerca en Sanitat Animal (CReSA), UAB-IRTA, Campus de la Universitat Autònoma de Barcelona, Bellaterra, Cerdanyola del Vallès 08193, Spain; 2 Institute of Medical Virology, Justus-Liebig-University, Schubertstrasse 81, Giessen D-35392, Germany; 3 Departament de Sanitat i Anatomia Animals, Universitat Autònoma de Barcelona, Bellaterra, Barcelona 08193, Spain; 4 Institut de Recerca i Tecnologia Agroalimentàries (IRTA), Barcelona 08007, Spain; 5 Institute of Virology, Philipps University Marburg, Hans-Meerwein-Strasse 2, Marburg 35043, Germany; 6 Centre de Recerca en Agrigenòmica (CRAG), Consorci CSIC-IRTA-UAB-UB, Campus UAB, Bellaterra, Cerdanyola del Vallès 08193, Spain; 7 Departamento de Patología, Escuela Medicina Veterinaria, Universidad Nacional Autonoma, Apartado 86, Heredia 3000, Costa Rica; 8 Department of Microbiology and Immunology, University of Melbourne, Parkville, Melbourne, VIC 3010, Australia

## Abstract

Some outbreaks involving highly pathogenic avian influenza viruses (HPAIV) of subtypes H5 and H7 were caused by avian-to-human transmissions. In nature, different influenza A viruses can reassort leading to new viruses with new characteristics. We decided to investigate the impact that the NS-segment of H5 HPAIV would have on viral pathogenicity of a classical avian H7 HPAIV in poultry, a natural host. We focussed this study based on our previous work that demonstrated that single reassortment of the NS-segment from an H5 HPAIV into an H7 HPAIV changes the ability of the virus to replicate in mammalian hosts. Our present data show that two different H7-viruses containing an NS-segment from H5–types (FPV NS GD or FPV NS VN) show an overall highly pathogenic phenotype compared with the wild type H7–virus (FPV), as characterized by higher viral shedding and earlier manifestation of clinical signs. Correlating with the latter, higher amounts of IFN-β mRNA were detected in the blood of NS-reassortant infected birds, 48 h post-infection (pi). Although lymphopenia was detected in chickens from all AIV-infected groups, also 48 h pi those animals challenged with NS-reassortant viruses showed an increase of peripheral monocyte/macrophage-like cells expressing high levels of IL-1β, as determined by flow cytometry. Taken together, these findings highlight the importance of the NS-segment in viral pathogenicity which is directly involved in triggering antiviral and pro-inflammatory cytokines found during HPAIV pathogenesis in chickens.

## Introduction

In recent decades highly pathogenic avian influenza viruses (HPAIV) belonging to H5– and H7- subtypes have been reported to cause major outbreaks in birds worldwide [1]. The overall impact in the poultry industry has dramatically increased in the last decade, from 23 million birds affected between 1959 and 1998 to more than 200 million, between 1999 and 2004 [2]. Besides their economic consequences, influenza A viruses (IAV) are currently considered as one of the most important threats to human health because of their pandemic potential, underlining the relevance of avian reservoirs for IAV. Since 2003, as confirmed by the World Health Organisation, transmissions of the H5N1 HPAIV to humans have caused approximately 628 disease cases and 374 deaths [3]. More recently, last March 2013, a novel influenza A/H7N9 virus was identified in humans. The A/H7N9 virus originates from multiple reassortment events of influenza viruses of avian origin; ducks and chickens have been suggested as intermediate hosts which led to the emergence of A/H7N9 [4].Therefore, it is important to increase our knowledge concerning the role of viral determinants in virulence and in different species to improve vaccine design and therapies against IAV.

The innate immune response is the first unspecific barrier of the host against pathogens, and the induction of type I interferon (IFN) expression, mainly IFNα/β, is one of the earliest anti-viral cytokines expressed upon IAV infection [5]. Although hosts develop antiviral responses in order to control the infection, IAV have evolved multiple strategies to avoid these responses [6]. By expressing the nonstructural protein 1 (NS1), IAV antagonize the immune response of infected cells, especially limiting the production of type I IFN, as well as that of other immunomodulators [7]. IFN-β induction can be limited by the NS1 protein by both pre-transcriptional (cytoplasmatic) and post-transcriptional (nuclear) processes [8,9]. However, the mechanisms and targets for NS1 depend on virus strain [9,10]. Furthermore, it has been demonstrated that the NS1 protein prevents virus-mediated activation of the following transcription factors: interferon regulatory transcription factor (IRF)-3 [11], nuclear factor kappa-light-chain-enhancer of activated B cells (NFκB) [12] and c-Jun/ATF-2 [13], which are essential for IFN-β induction. The contribution of the NS1 protein to the pathogenicity of IAV has been demonstrated in mammalian models, such as mice and pigs [14-16]. Although less is known in avian species, Li et al. reported that, as in mammals, in both chickens and geese NS1 has an important role in viral virulence [17]. Many functions of the NS2 protein, which is translated from the eighth RNA segment (the same genome segment as for the NS1 protein), are, however, not understood. Its role in mediating the export of vRNP from the nucleus to the cytoplasm is well studied [18].

Pro-inflammatory cytokines secreted during IAV infection have also been described to have an important role in mammalian species [19,20]. In a mouse model, lymphocyte apoptosis and high-level inductions of cytokines, including interleukin 1 (IL-1), have been proposed to contribute to the severity of IAV [21]. Little is known, however, about the role of IL-1 in chickens, the natural host of IAV. Thus, further studies about the role of pro-inflammatory cytokines in disease severity and outcomes in chickens infected with HPAIV are needed.

The present study was designed to determine the impact that an NS-segment reassortment between two HPAIV would have on the pathogenicity of IAV. The role of the NS-segment was assessed using a recombinant H7N1 virus (A/FPV/Rostock/34; FPV) with NS segments from H5N1 viruses: A/Goose/Guangdong/1/96 (GD) or A/Viet Nam/1203/2004 (VN) previously generated by reverse genetics. The new viruses are named FPV NS GD and FPV NS VN, respectively. We show that NS segments from the H5-type HPAIV can alter the pathogenicity of FPV in chickens by increasing clinical manifestations. Interestingly, the exchange of NS segments also modifies the host antiviral response, as demonstrated by an increase in IL-1β production in both FPV NS- GD and -VN infected chickens, as early as 2 days after infection.

## Materials and methods

### Cell culture and viruses

Madin-Darby Canine Kidney (MDCK) cells were purchased from ATCC (CCL-34) and cultured according to the manufacturer’s instructions in Dulbecco’s modified Eagle’s medium (DMEM) (Life Technologies, S.A, CA, USA) supplemented with 10% fetal calf serum (FCS) and antibiotics (100 U mL^-1^ penicillin and 0.1 mg mL^-1^ streptomycin) at 37 °C in a 5% CO_2_ humidified atmosphere.

Viruses used in this study were the A/FPV/Rostock/34 (FPV; H7N1) generated by recombinant technology and two reassortants carrying the NS-segment of either A/Goose/Guangdong/1/96 (GD; H5N1) or A/Viet Nam/1203/2004 (VN; H5N1) in the genetic background of FPV. The reassortants are named FPV NS GD and FPV NS VN, respectively. All these viruses were generated in the facilities of the Institute of Medical Virology at Justus-Liebig-University in Giessen (Germany), as previously described [14,22]. Virus stocks were propagated in the allantoic cavity of 11-day-old embryonated chicken specific-pathogen-free (SPF) eggs (Lohmann Tierzucht GmbH, Cuxhaven, Germany) at 37 °C for 72 h. The allantoic fluids were harvested, aliquoted and stored at −80 °C until use. Virus titer was determined in 11-day-old embryonated chicken SPF eggs and measured as egg lethal doses 50% (ELD_50_) according to the Reed and Muench method [23].

### Animal experiments

Ninety-five SPF-eggs (Lohmann Tierzucht GmbH) were hatched under BSL-3 containment conditions at the *Centre de Recerca en Sanitat Animal* (CReSA-Barcelona). Two-week-old chickens were divided into four groups and placed in independent negative pressure isolators. The animals were inoculated intranasally with 10^5.5^ ELD_50_ of either FPV (Group 1, *n* = 25), FPV NS GD (Group 2, *n* = 25) or FPV NS VN (Group 3, *n* = 25) in a volume of 50 μL. One group of twenty chickens was mock-infected with PBS 1× in a volume of 50 μL and used as a negative control (Group 4). Chickens were monitored for clinical signs and the mean clinical score, as well as mortality rate, were recorded. Intensity of clinical signs was assessed by a semi-quantitative scoring indicating: healthy (0), sick (1) or severely sick (2). A scoring of (1) was given to those animals showing one of the following signs, and (2) to those showing more than one of the following signs: respiratory involvement, depression, diarrhoea, cyanosis of the exposed skin or wattles, oedema of the face and/or head, nervous signs. According to the ethical procedures, the animals were euthanized with intravenous administration of sodium pentobarbital (100 mg/kg) if severe clinical symptoms became apparent.

Ten animals per group were kept to describe the clinical outcome and mortality rate. The other chickens (*n* = 15/group) were used to obtain samples and to perform necropsies. From these animals, blood samples were obtained from three chickens from each group at 6 h post infection (pi) and 1, 2, 3 and 4 days post infection (dpi). Blood was collected from the heart after anaesthetizing the animals with Zoletil® (Virbac, Carros cedex, France). Two to four mL of blood were collected in tubes containing 2 mL of Alsever’s anticoagulant (Sigma-Aldrich, Madrid, Spain). Oropharyngeal and cloacal swab samples (OS and CS, respectively) were collected at the same time pi in DMEM containing antimicrobial drugs (100 U mL^-1^ penicillin and 0.1 mg mL^-1^ streptomycin). Two animals from Group 4 (negative control) were also sampled at the same time points as the other groups.

The present study was performed in strict accordance with the Guidelines of Good Experimental Practices. Animal procedures were approved by the Ethical and Animal Welfare Committee of *Universitat Autònoma de Barcelona* (UAB) (Protocol #DMAH-5767). Chicken experiments were conducted at Biosafety Level 3 (BSL-3) facilities of CReSA.

### Histopathology and viral nucleoprotein antigen determined by immunohistochemistry

Necropsies and tissue sampling were performed according to a standard protocol. For histopathological analysis, collected tissues were fixed in 10% neutral buffered formalin, dehydrated and embedded in paraffin. Tissue microtome sections (4 μm) were processed routinely for hematoxylin/eosin (H/E) staining and immunohistochemistry (IHC) for the detection of type A influenza virus NP. The following tissues were examined: central nervous system (CNS), heart, kidney, pancreas, liver, spleen, thymus and bursa of Fabricius.

An IHC technique based on the avidin-biotin complex immunoperoxidase system was performed as previously reported [24]. Briefly, a mouse-derived monoclonal commercial antibody against the nucleoprotein (NP) of influenza A virus (IgG2a, HB65, ATCC) was used as a primary antibody. As a secondary antibody, a biotinylated goat anti-mouse IgG antibody (GaMb, Dako E0433, Denmark) was used. Finally, an avidin-biotin-peroxidase complex (Thermo Fisher Scientific, Rockford, IL, USA) was used and the reaction was developed with 3,3′-Diaminobenzidine tetrahydrochloride (DAB) (brown colour) (Sigma-Aldrich) at room temperature (RT). Sections were counterstained with Mayer’s haematoxylin. Negative controls were those tissues from sham-inoculated animals (Group 4) and also tissues incubated without the primary antibody. Tissues from previous experiments demonstrated to be positive against NP by IHC were used as positive controls. To measure the extension of the staining in tissues a semi-quantitative scoring was assessed as follows: no positive cells (−), single positive cells (+), scattered groups of positive cells (++), and widespread positivity (+++).

### Virus quantification by real time RT-PCR (RRT-qPCR)

Viral RNA quantification using one step RRT-qPCR was performed in blood and OS and CS. Viral RNA was first extracted with Trizol (Life Technologies, S.A) obtaining 60 μL of eluted viral RNA, as described by the manufacturer. Briefly, after 2–3 min incubation with 0.2 mL of chloroform, samples were centrifuged at 12 000 × *g* for 15 min at 4 °C. When the aqueous phase was removed and placed into a new tube, 0.5 mL of 100% isopropanol were added and incubated 10 min at RT. After centrifugation at 12 000 × *g* for 10 min at 4 °C, the RNA was washed with 1 mL of 75% ethanol, centrifuged at 7500 × *g* for 5 min at 4 °C and air dried for 10 min. The RNA was re-suspended in DEPC-water and stored at −80 °C until use.

Amplification of a matrix (*M*) gene fragment was carried out using primers, probe, One-Step RT-PCR Master Mix Reagents (Life Technologies, S.A) and amplification conditions as described previously by Busquets et al. [25] in Fast7500 equipment (Life Technologies, S.A) using 5 μL of eluted RNA in a total volume of 25 μL.

### Cytokine quantification by real-time RT-PCR (RRT-qPCR)

Total RNA from the blood of three chickens per group and per time point (6, 24 and 48 h pi) was isolated using Trizol (Life Technologies, S.A.), as described in the previous section. The isolated RNA was reverse-transcribed into cDNA using the High Capacity cDNA Reverse Transcription kit (Life Technologies, S.A) according to the manufacturer’s instructions.

Primers and probes for IL-1β and for the housekeeping gene 28S, designed by Kaiser et al. [26] were used. For IFN-β, primers and probe were designed for the IFN-β gene sequence available at GenBank (NM_001024836). The probe was labelled with the fluorescent reporter dye 5-carboxyfluorescein (FAM) at the 5′-end and with the quencher N,N,N,N’-tetramethyl-6-carboxyrhodamine (TAMRA) at the 3′-end [5′-(FAM^*^)-CGCATCCTCCAACACCTCTT-(TAMRA)-3′].

Amplification and detection of specific products were performed using the TaqMan Universal PCR Master Mix (Life Technologies, S.A) with the following cycle profile: one cycle of 50 °C for 2 min, 95 °C for 10 min and 40 cycles of 95 °C for 15 s, 60 °C for 1 min, in Fast7500 equipment (Life Technologies, S.A). The mRNA expression level was calculated using the 2^-∆∆Ct^ method [27]. The results are expressed as fold change in comparison to a calibrator sample (samples from non-infected chickens, Group 4).

### Isolation of mononuclear cells

Peripheral blood mononuclear cells (PBMC) were isolated from whole blood by density-gradient centrifugation using Histopaque®-1077 (Sigma-Aldrich). The instructions from the manufacturer were followed with some modifications. Briefly, 4 mL of Histopaque-1077 were overlaid with 4 mL whole anticoagulated-blood and centrifuged for 20 min at 400 × *g* at RT. Following centrifugation, the opaque interface was collected and washed twice with 1 mL PBS 1× solution and centrifuged again for 10 min at 250 × *g*. Cell numbers were calculated using a dye solution and the cell concentration was adjusted to 10^7^ cells/mL.

### Flow cytometric analysis

Flow cytometry studies allowed separating the blood subpopulations by size and complexity (FSC and SSC, respectively); therefore, distinguishing lymphocytes, monocytes and macrophages from histiocytes and heterophils [28]. An allophycocyanin (APC) Antibody Conjugation Kit (Bionova Cientifica, Madrid, Spain) was used to conjugate both IL-1β and IFN-β purified mAb, according to the manufacturer’s instructions. Approximately 10^6^ PBMC per well were added in V-bottomed 96-well plates. The cells were fixed (PBS + 2% PFA) during 15 min, washed with FACS diluent (PBS + 2% FCS) and finally, the cells were permeabilized with 200 μL of diluted detergent (saponin). Following two washes with FACS diluent, 50 μL of either mAb IL-1β APC-conjugated or IFN-β APC-conjugated diluted with FACS diluent were added and incubated for 20 min. All procedures were carried out at 4 °C. Analyses were done using a BD FACSAria I Flow Cytometer (Becton Dickinson, NJ, USA).

### Statistical analysis

**Figure 1:**
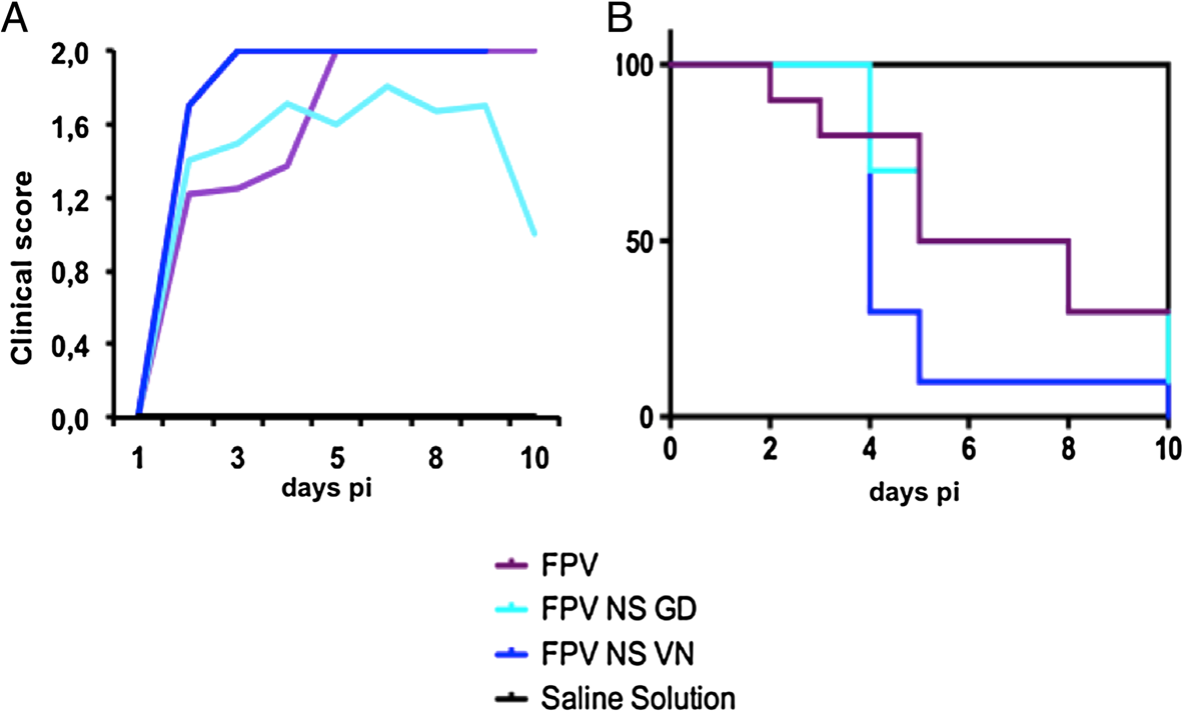
**Clinical score and survival rate of SPF-chickens after infection with HPAIV.** Chickens were challenged with 10^5.5^ ELD_50_ of FPV, FPV NS GD or FPV NS VN. *n =* ten chickens per group*.* Average clinical signs of the surviving chickens **(A)** and survival rate **(B)** from each group measured at different time-points after infection. The results were analyzed with the Kaplan-Meier method and the survival curves were compared by the log-rank and the Gehan-Breslow-Wilcoxon tests. No statistical differences were observed between the survival curves.

The results correspond to the Mean ± Standard Error of the Mean (SEM) of the indicated experiments. Differences between groups were tested for significance using the Student’s *t* test. Differences were considered statistically significant at *P* < 0.05. Statistical analyses were performed using SPSS for Windows, version 17.0. The results from Figure 1 were analyzed with GraphPad Prisme 6 software, using the product limit method of Kaplan-Meier, and comparing survival curves using both the log-rank test and the Gehan-Breslow-Wilcoxon test.

## Results

### NS segment from H5-type viruses increases the virulence and shedding of H7N1 HPAIV in chickens

Although FPV is not pathogenic in mice [29,30], in chickens it is considered a highly pathogenic virus [31], as also confirmed in the present study. FPV infection resulted in the appearance of severe clinical signs between 5 and 7 dpi (Figure 1A), 50% mortality by day 5 pi and 70% of mortality rate by the end of the experiment, at day 10 pi (Figure 1B). Unexpectedly, the clinical signs found in FPV NS GD infected chickens did not increase dramatically, showing very similar disease outcome and death kinetics when compared with FPV (Figure 1A) at the early time-points, albeit the final mortality rate was higher, with 90% of the infected animals being dead by day 10 pi. A more dramatic effect was observed for the FPV NS VN reassortant virus, causing more severe disease than the other two viruses, as early as 2–3 dpi (Figure 1A), with 90% of the deaths recorded 5 dpi and 100% mortality by day 10 pi (Figure 1B). However, no statistical differences were observed when comparing the mortality rates between groups.

Clinical signs shown in all groups were non-specific and consisted of depression, apathy, mucous nasal discharge and ruffled feathers. More severe neurological clinical signs as torticollis and lack of coordination were also observed. Gross lesions were observed as early as 2 dpi in FPV NS VN and started between 3 and 4 dpi in FPV and FPV NS GD groups. The lesions were similar between groups and consisted of conjunctivitis, multifocal to diffuse haemorrhages, cyanosis of the comb and diffuse oedema. At necropsy, petechial haemorrhages on leg muscles, breast and serosa of the proventriculus were detected in all the infected groups. From 3 to 7 dpi, splenomegaly and atrophy of both the thymus and bursa of Fabricius were observed in FPV NS GD and FPV NS VN groups (data not shown).

Tissue samples were fixed with 10% formalin and embedded in paraffin for histopathological analysis. Tissue sections were stained with H/E and avian influenza virus (AIV)-NP was detected by IHC (Table 1, Additional file 1). Evaluation of H/E sections from chickens sacrificed both at 6 h pi and 1 dpi revealed no tissue damage in comparison to the non-infected group. Nervous system lesions were detected as early as 3 dpi in all groups, being more extensive in both the NS reassortant-infected groups. The lesions in CNS consisted of multifocal areas of malacia associated with immune staining on neurons and glial cells (Table 1). Multifocal areas of myocardial necrosis were observed in birds mainly from FPV NS GD and FPV NS VN groups from 3 dpi. The positivity to the AIV antigen observed in myocytes correlated with this finding. In FPV-infected animals these lesions were first observed at day 4 pi and only in one animal. However, one bird showed AIV-positive myocytes 3 dpi. In all groups, slight lesions were observed in the liver with Kupffer’s cells showing positivity for viral antigen (Table 1). Moderate lesions were observed in the kidney of FPV-infected birds, while more severe lesions were observed in the reassortant-groups, consisting of areas of tubular necrosis associated with the presence of viral antigen. Infiltration of heterophils and macrophages were found in the necrotic areas. Severe lesions were also observed in the pancreas, which from 3 dpi on showed diffuse areas of necrosis and stained for the presence of the associated viral antigen (Table 1). Concerning the lymphohematopoietic organs studied (thymus, spleen and bursa of Fabricius) we observed moderate to severe lymphoid depletion from day 2 pi on.

**Table 1:** Average distribution of AIV-nucleoprotein antigen determined by immunohistochemistry (IHC) in tissue samples

**VIRUS STRAIN**	**Time of AIV NP antigen detection**				**Main localization**
**Tissue**	**1 dpi**	**2 dpi**	**3 dpi**	**4 dpi**	
FPV					
CNS	-	-	+	+	Neurons, glial cells
Heart	-	+	+	+	Myocytes
Kidney	-	-	−/+	+	Epithelialtubularcells
Pancreas	-	-	+	+	Exocrine acinar cells
Liver	-	-	+	+	Kupffer’s cells
Spleen	-	-	+	+	Macrophages
Thymus	-	+	+	+	Macrophages
Bursa of Fabricius	-	-	+	+	Macrophages
FPV NS GD					
CNS	-	-	++	+	Neurons, glial cells, ependymalcells
Heart	-	++	+	+	Myocytes, macrophages
Kidney	-	+++	++	++	Epithelial tubular cells
Pancreas	-	-	++	+	Exocrine acinar cells
Liver	-	-	+	+	Kupffer’s cells
Spleen	-	+	+	+	Macrophages, Endothelial cells
Thymus	-	+	+	+	Macrophages, Endothelial cells
Bursa of Fabricius	-	-	+	+	Macrophages, Endothelial cells
FPV NS VN					
CNS	-	-	++	+	Neurons, glial cells, ependymalcells
Heart	-	++	+	+	Myocytes, macrophages
Kidney	-	-	++	++	Epithelial tubular cells
Pancreas	-	-	++	+	Exocrine acinar cells
Liver	-	-	+	+	Kupffer’s cells
Spleen	-	-	+	+	Macrophages, Endothelial cells
Thymus	-	-	+	+	Macrophages, Endothelial cells
Bursa of Fabricius	-	-	+	+	Macrophages, Endothelial cells
Saline Solution					
CNS	-	-	-	-	-
Heart	-	-	-	-	-
Kidney	-	-	-	-	-
Pancreas	-	-	-	-	-
Liver	-	-	-	-	-
Spleen	-	-	-	-	-
Thymus	-	-	-	-	-
Bursa of Fabricius	-	-	-	-	-

Chickens were inoculated with FPV, FPV NS GD or FPV NS VN and necropsies were performed at different time-points. The extension of the staining in tissues was measured by a semi-quantitative score: no positive cells (−), single positive cells (+), scattered groups of positive cells (++), and widespread positivity (+++).

**Figure 2:**
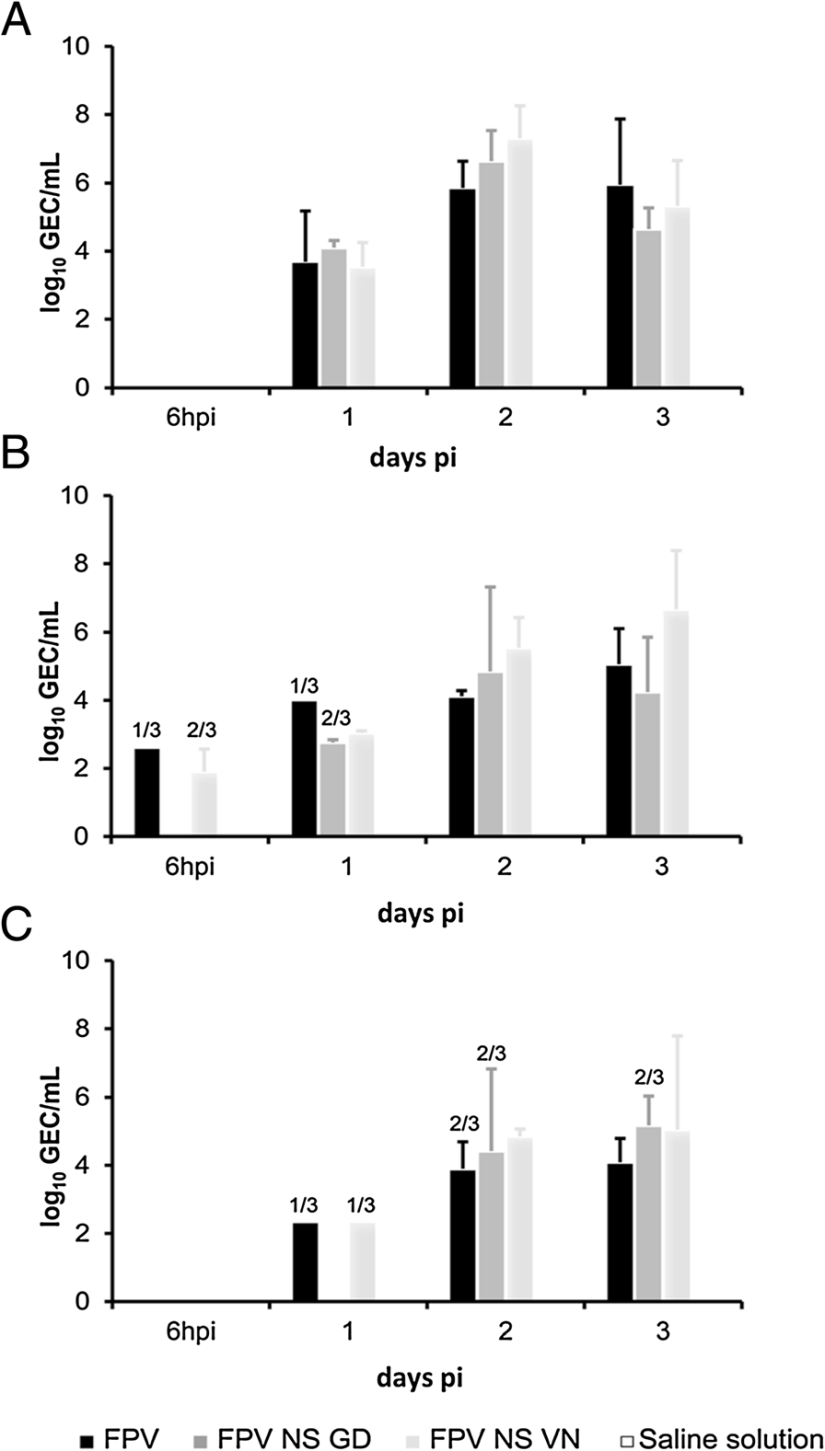
**Detection of influenza viral RNA after infection with FPV, FPV NS GD or FPV NS VN.** Quantification of influenza viral RNA was performed by RRT-PCR in blood **(A)** oropharyngeal swab **(B)** and cloacal swab **(C)** at the time points indicated. The results are expressed in genome equivalent copies (GEC) of plasmid per sample and shown as means ± SD. In the case that not all the samples from a group (3/3) are positive, the number of positive samples from the total number of animals is indicated above each bar. Statistical significant differences (*p* < 0.05) are indicated with letters. Different letters indicate statistical difference between groups and the same letter means that no statistical difference was observed. Samples from animals in the negative group (G4) were all below the limit of detection.

The viraemia and the shedding of the three viruses was characterized by determining the amount of viral RNA present in the blood (Figure 2A) and OS (Figure 2B) and CS (Figure 2C) at early time points post infection. A quantitative real time RT PCR was performed from 6 h pi until day 3 pi. Animals infected with FPV NS VN showed significantly higher presence of viral RNA in blood and in OS at 2 dpi compared with the FPV-group (*P* < 0.05). In the FPV NS GD group, viraemia and shedding were also higher compared with the FPV-group, but the differences were not statistically significant when compared between groups. No differences were observed for both NS-reassortant groups in any of the time points.

### Comparison of the transcription and expression of IL-1β and IFN-β genes in infected-chickens

Antiviral and pro-inflammatory cytokine expressions (IFN-β and IL-1β as representative cytokines) in the blood were sequentially studied throughout the infection. We performed a relative RT-qPCR to study the changes in IFN-β and IL-1β mRNA transcription after the infection with IAV, using the 28S gene as the housekeeping gene.

**Figure 3:**
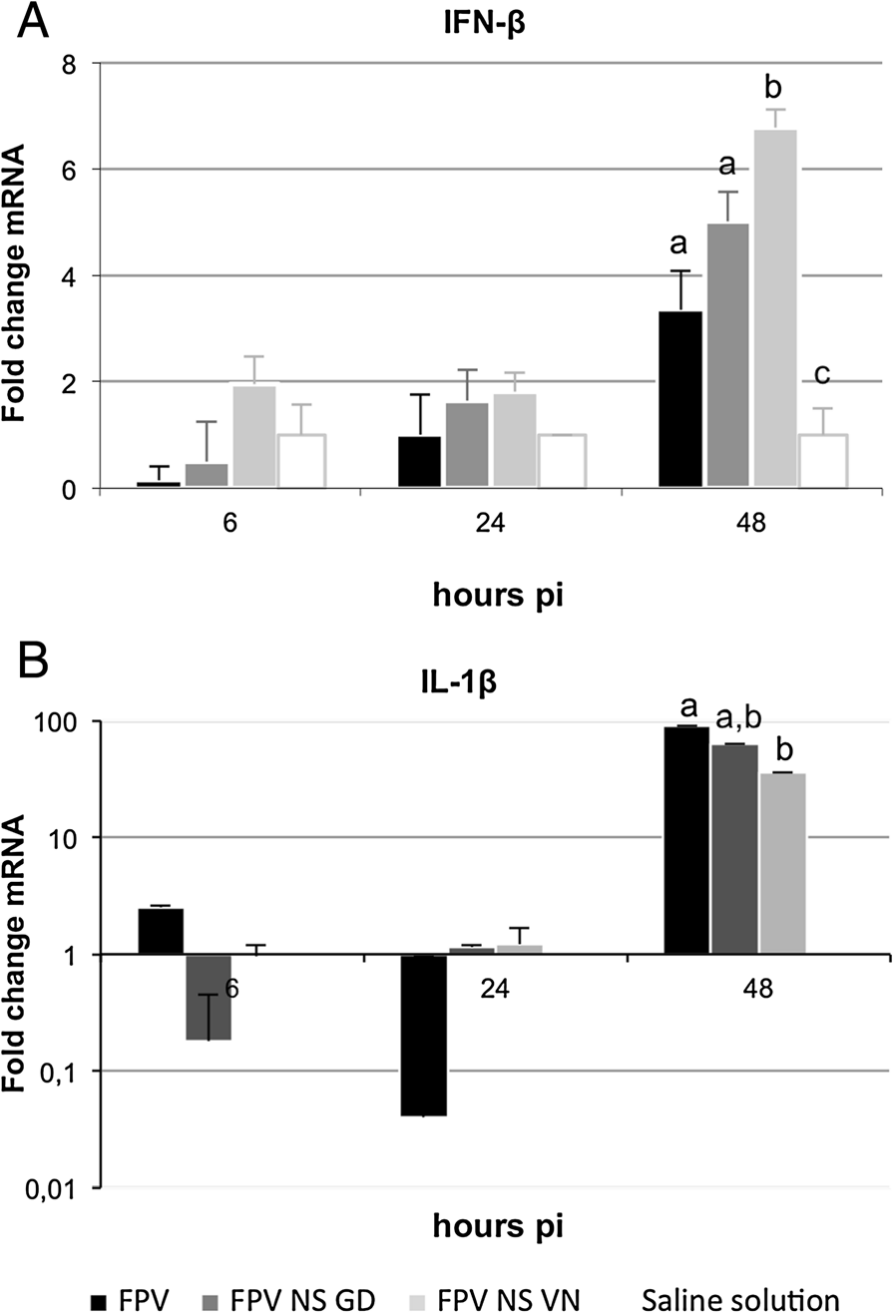
**Quantification of IFN-β and IL-1β gene expression in PBMC after FPV, FPV NS GD or FPV NS VN infection.** The cytokines IFN-β **(A)** and IL-1β **(B)** mRNA gene expression profile of PBMC were analyzed by quantitative RT-PCR. The results are expressed as means ± SD from three experiments. Statistical significant difference (*p* < 0.05) is indicated with letters. Different letters indicate a statistical difference between groups and the same letter means that no statistical difference was observed.

At 48 h pi, different levels of IFN-β gene transcription were observed between groups (Figure 3A). At this time-point, the levels of IFN-β mRNA were higher in FPV NS VN-infected animals, coinciding with the disease exacerbation observed. At the earlier time-point no evident differences were detected between groups. A significant differential IL-1β gene expression was also observed between inoculated and non-inoculated groups at 48 h pi (*P* < 0.05; Figure 3B), showing higher IL-1β mRNA levels in the former. In contrast with that observed for IFN-β, FPV-infected animals showed a higher up-regulation of IL1-β compared with those infected with the NS-recombinants. Interestingly, slight down-regulations of IL1-β were observed in FPV- and FPV NS GD-blood at 24 and 6 h pi, respectively, albeit these differences were not statistically significant (Figure 3B).

### IL1-β upregulation correlates with an increase in monocyte/macrophage-like cells

**Figure 4:**
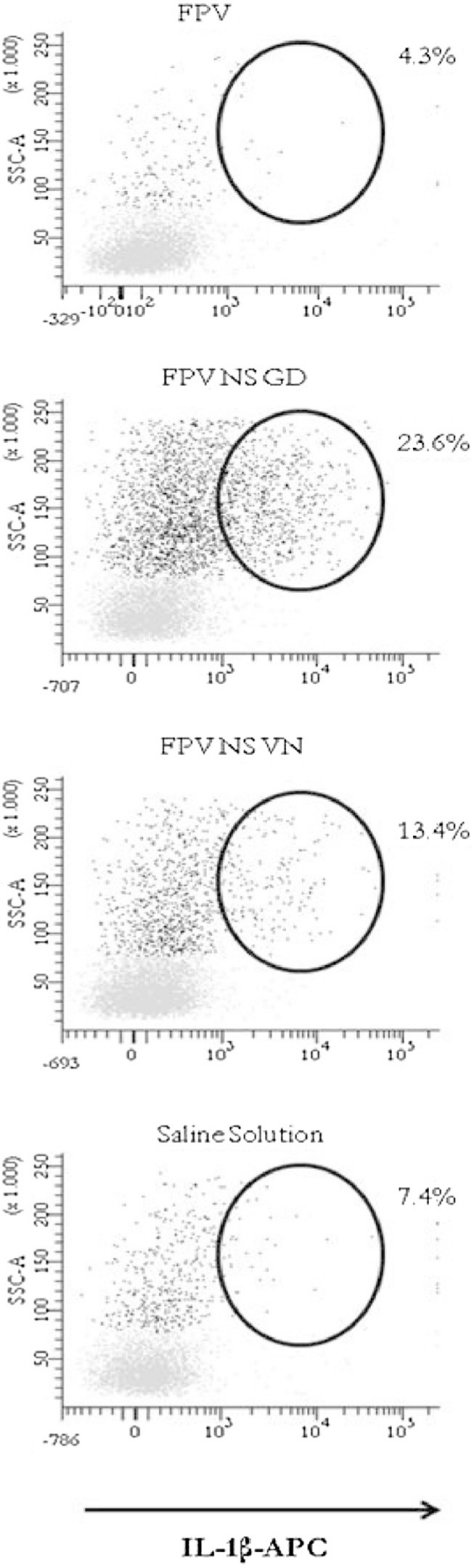
**Expression of IL-1β protein induced the following infection with FPV, FPV NS GD or FPV NS VN in PBMC measured by flow cytometry.** Flow cytometry of PBMC of chickens at day 2 after infection with the influenza virus. The black population represents monocyte/macrophage-like cells and the lymphocyte population is shown in light grey. The numbers above the outlined areas indicate the percent of the total population of IL-1β single expressing-cells.

Biological processes to external perturbations are regulated by a complex mechanism inside cells. To understand these processes in our in vivo model we integrated the data obtained by measuring the levels of mRNA of both IL-1β and IFN-β cytokines (Figure 3), and data from the protein measurement (Figure 4) in blood samples.

To test the amount of synthesized protein, as well as the cell population distribution after the infection, a flow cytometry analysis was performed, allowing us to separate the blood populations by size and complexity. Independently of the individual variability, blood from NS-reassortant virus infected chickens clearly showed a lymphocyte reduction that correlated with an increase in the number of macrophage/monocyte-like cells that peaked at 48 h pi Interestingly, while no evident up-regulation of IFN-β was observed independently of the animal group and infection time (data not shown), a dramatic increase of IL-1β expression was found in both reassortant-infected groups at 48 h pi, being higher in those animals infected with FPV NS GD (23.6% cells expressing IL-1β) (Figure 4). In contrast, at 48 h pi, chickens infected with FPV exhibited a slight decrease (4.3%) in the total population of IL-1β single expressing-cells compared to the non-infected group (Figure 4). Thus, NS-reassortant infected-chickens showed clinical signs from day 2 pi, whereas in FPV-infected ones the severe clinical signs (score = 2) were observed from day 5 pi (Figure 1).

## Discussion

The pathogenic potential of some strains of IAV has been related to multiple factors, including viral determinants and an excessive host immune response [32-41]. Since most of these descriptions have been done in mammals, in the present study we tried to extend these studies to natural hosts of AIV. More specifically, we focused our studies on the understanding of the role that the AIV NS-segment of two different H5- HPAIV strains plays during pathogenesis in birds. To this end, an experimental infection in SPF-chickens was designed with an H7N1 HPAIV (FPV) and two reassortants carrying the NS-segment of H5N1 HPAIV from either GD (FPV NS GD) or VN (FPV NS VN).

Amino acid changes in the viral hemagglutinin and polymorphisms in the polymerase subunit have been shown to contribute to the virulence of AIV [42]. However, among other viral proteins, NS1 is one of the major pathogenicity factors and it mainly acts by suppressing type I IFN-activities [8], which are the first line of host defence against viral infections. The role of AIV-NS1 in pathogenesis has been investigated for decades, albeit most of this work has been focused on understanding its role in mammalian species. Treanor et al. reported that efficient infection by FPV depends on the NS1 allele incorporated within its genome [43]; a fact more recently confirmed by demonstrating that specific H5N1 NS-reassortants increase the viral replication ability in mice and a host range of FPV [14,22]. Here, these studies have been extended to SPF-chickens, an ideal animal model to study AIV. We show that both NS-reassortants (FPV NS GD and FPV NS VN), which differ from the FPV in the NS segment, also demonstrates increased virulence in chickens. However, due to the fact that FPV is an HPAIV strain in chickens [31], the differences observed in this species were not as dramatic as those shown before in mice, where this virus is non-pathogenic [14]. Therefore, we could conclude that apart from its role in overcoming species-specific barriers, AIV-NS also accomplish a very important role as a virulent factor in avian hosts. The differences observed in the in vivo pathogenesis of the FPV, FPV NS GD and FPV NS VN seem to correlate with the differential level of NS1 protein expression, at least as shown by in-cell Western blot using MDCK cell extracts infected with the different recombinant viruses [22].

Amino acid differences or substitutions in the NS1 protein are described to alter its subcellular localization [44,45]. This was recently confirmed for the NS1-sequences for the viruses used here [14,22]. As such, the comparison of the NS1 amino acid sequences of the viruses used in the present study showed that the residues 217 and 221 in the nuclear localisation signal 2 (NLS2) of FPV NS VN were different or deleted, respectively, compared to the NLS2 of FPV and FPV NS GD. Moreover, the nuclear export signal (NES) of FPV NS GD presents some amino acid differences compared to the other two viruses. An additional Figure shows this in more detail (Additional file 2). The differences found, affecting either NLS2 and/or NES, could explain the differential localization of each NS1 within the infected cell, with the FPV-NS1 being found in the nucleus of the cell, the GD-NS1 being localized within the cytoplasm and with the VN-NS1 occupying both intracellular locations [22,46,47]. This differential localization could also explain the different modulation observed for both cytokines, IFN-β and IL-1β at the transcriptional (mRNA) and post-transcriptional (protein) level.

However, while the impact of NS1 variants in the pathogenesis of AIV in the chicken seems to fit perfectly well with previous studies described in the literature, their correlation with the IFN expression levels did not seem to fit that well, at least as shown at late times post-infection. Thus, at 48 h pi the reassortant viruses show a similar upregulation of IFN as the FPV virus. On the one hand, most of the previous results described for the impact of NS1 in IFN antagonism come from in vitro studies done with established cell lines or in vivo using the mouse model. Agreeing with this assumption, it has been shown that highly pathogenic avian influenza viruses do not inhibit interferon synthesis in infected chickens [48]. On the contrary, the IFN upregulation shown in our study was only detectable at 48 h pi and at an RNA level, perhaps reflecting the systemic dissemination of the virus. The fact that the levels of IFN expression remain at a basal level at 6 and 24 h pi seems to confirm the hypothesis of a real downregulation of IFN at early time post-infection, when innate immunity should be totally operative.

Interestingly, a very strong overexpression of IL-1β was observed after infection with the GD and VN NS-reassortants, detectable even in the absence of any in vitro re-stimulation. The over expression of IL-1β found after the infection with the NS-reassortants might obey an increase of the virus replication in monocyte-like cells and macrophage-like cells, rather than a direct effect of the different NS-segments.

In fact, all samples obtained from FPV infected chickens showed lower levels of IL-1β positive-cells (<10%) than those obtained from chickens infected with the NS-reassortant viruses. On the contrary to that described for mice [29,30] all viruses used were highly pathogenic in the chicken, making the comparative studies more challenging. This, together with the fact that the assay was only performed using samples from one time-point infected chickens (48 h pi), perhaps not optimal for all AIV strains, could help to explain the lack of concordance found between the percentage of IL-1β positive-cells and virulence for the GD- and VN- reassortants. Future quantifications of IL-1β positive-cells should also take into account the potential leucopenia suffered by the animals, a phenomenon that could be based on the in vivo significance of a given percentage of IL-1β positive-cells.

Independently of the differences found at the RNA level, no significant differences were observed regarding the expression of IFN-β, while significant differences were found for IL-1β, mainly at 48 h pi. Thus, the presence of the GD- or VN-NS segment seemed to enhance the induction of IL-1β expression, mainly by monocyte-like and macrophage-like cells (Figure 4). IL-1β plays a dual role for host immunity and together with IL-18 form what is named inflamasome, an essential innate mechanism that has to be activated in order to prime the immune system for future memory adaptive specific responses [49]. On the contrary, IL-1β by itself has been directly linked to autoimmune disorders and also to immunopathogenesis after infection with all kinds of pathogens including viruses. IL-1β is a key immunomodulator cytokine that plays a multifactorial role including two apparently opposite functions: (i) it forms part of what has been called an inflammasome (as mentioned above), an innate immune machinery that plays an essential role to mount adaptive immune responses against pathogens, including influenza viruses [50] and (ii) as an antipyretic. IL-1β can be elevated immediately after the infection with virulent avian virus strains as demonstrated for reovirus [51] or Marek infections [52] and also as a consequence of bacterial and virus co-infections, likely contributing to exacerbating lesions [53].

As the main conclusions, our work demonstrates the following: (i) that the NS segment from the H5N1 HPAIV has an impact on the FPV pathogenesis in chickens and (ii) that this increase in pathogenesis was coincident with an early over-expression of IL-1β from monocyte/macrophage-like cells.

These studies could be of utility to improve our understanding of the pathogenesis of HPAIV and to develop future antiviral strategies. For example, FPV NS GD or FPV NS VN could additionally be modified by deleting the RNA-binding motif of the NS1 protein; thus, obtaining replication-deficient, and much more immunogenic influenza virus vaccines than those previously described in the literature using this method [54].

## Competing interests

The authors declare that they have no competing interests.

## Authors’ contributions

JVA, RR and ZW prepared the viruses used in this study. JVA, AJC, RR, RD, NM and AD participated in the daily monitoring of the clinical signs and the sampling of animals. JVA, AJC, RD, and NM performed the necropsies and the tissue sampling. JVA and NM carried out the histopathological examinations. JVA, NB, MB and RR carried out the RRT-PCRs. NB, NM, FR and AD conceived the study and participated in its design and coordination. JVA, NB, MB, SP, FR and AD wrote the manuscript. All authors read and approved the final manuscript.

## Supplementary Material

Additional file 1 **Distribution of NP antigen in two representative tissues (heart and kidney) of chickens challenged with FPV, FPV NS GD, FPV NS VN or saline solution at day 3 pi.** Viral antigen was found in myocytes (heart, left panel) and in epithelial tubular cells (kidney, right panel) from all IAV-infected groups. Click here for file

Additional file 2 **Comparison of the NS1 of FPV (H7N1), GD (H5N1) and VN (H5N1).** Identical amino acids are boxed in black. The regions of the RNA-binding domain and the effector domain are underlined by dark blue and light blue bars, respectively. Click here for file
