# Supplementary figures and images for: The NS segment of H5N1 avian influenza viruses (AIV) enhances the virulence of an H7N1 AIV in chickens
Source: Vet Res. 2014 Jan 25;45(1):7. doi: 10.1186/1297-9716-45-7 (PMC3922795; doi:10.1186/1297-9716-45-7)

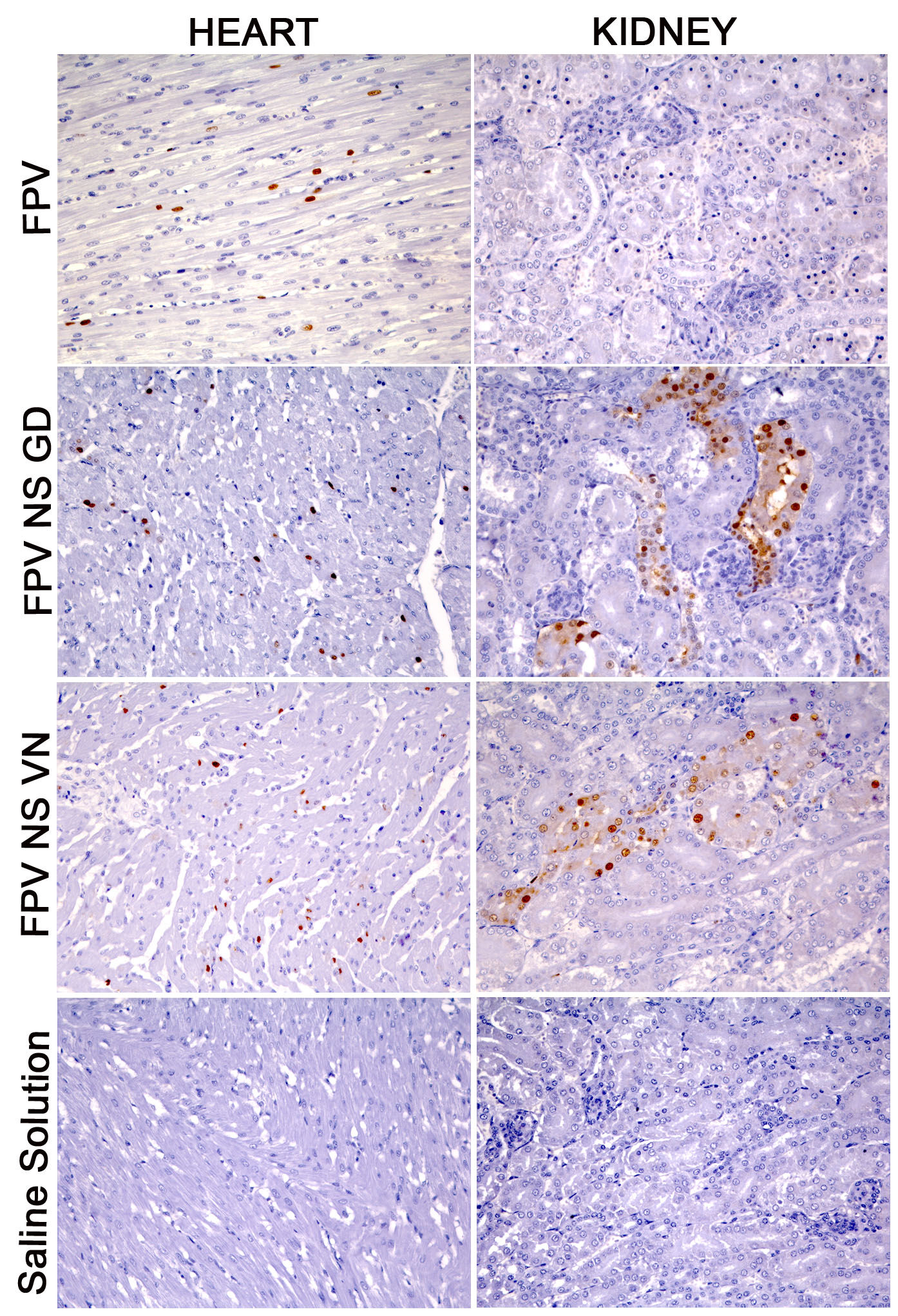

Supplement: Additional file 1 — Distribution of NP antigen in two representative tissues (heart and kidney) of chickens challenged with FPV, FPV NS GD, FPV NS VN or saline solution at day 3 pi. Viral antigen was found in myocytes (heart, left panel) and in epithelial tubular cells (kidney, right panel) from all IAV-infected groups. [file 1297-9716-45-7-S1.tiff]

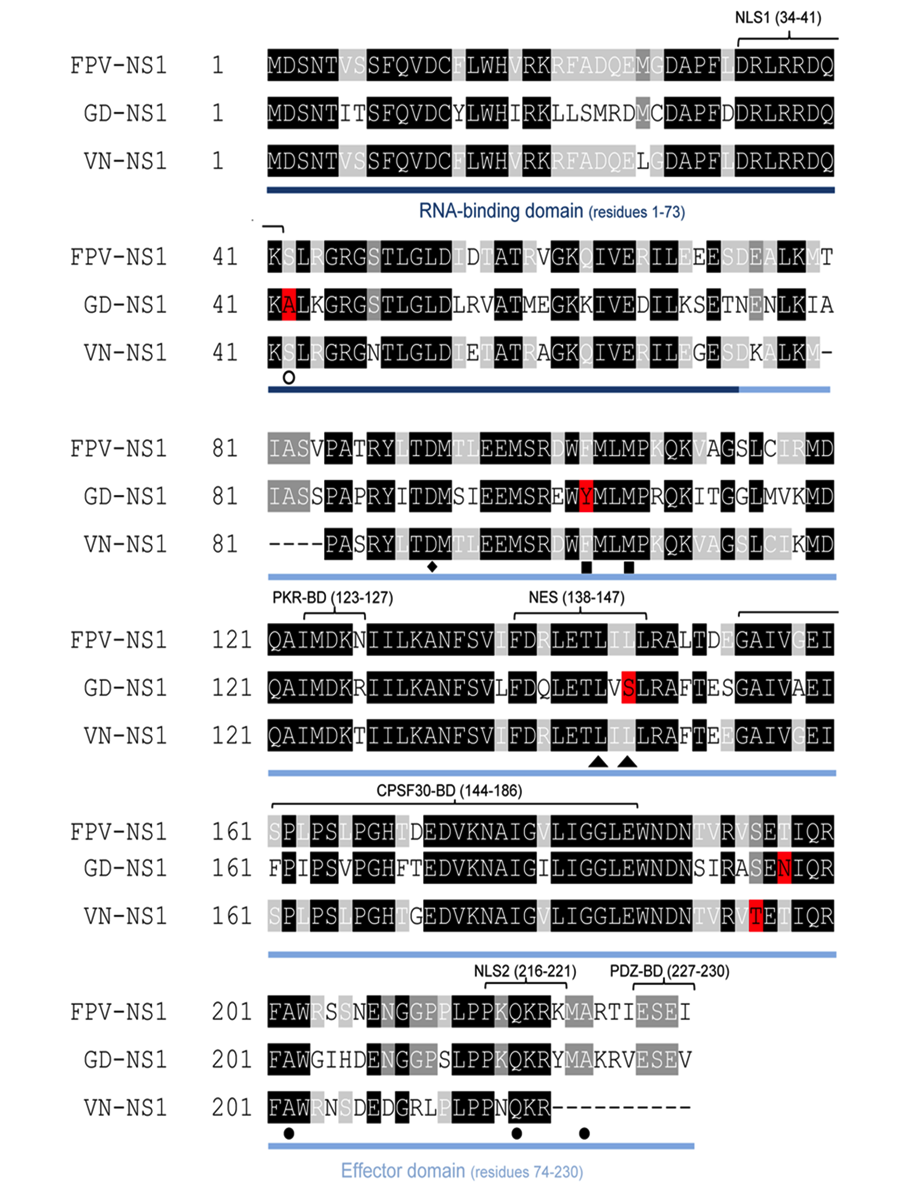

Supplement: Additional file 2 — Comparison of the NS1 of FPV (H7N1), GD (H5N1) and VN (H5N1). Identical amino acids are boxed in black. The regions of the RNA-binding domain and the effector domain are underlined by dark blue and light blue bars, respectively. [file 1297-9716-45-7-S2.tiff]
